# Synthesis of conformationally restricted glutamate and glutamine derivatives from carbonylation of orthopalladated phenylglycine derivatives

**DOI:** 10.3762/bjoc.8.179

**Published:** 2012-09-18

**Authors:** Esteban P Urriolabeitia, Eduardo Laga, Carlos Cativiela

**Affiliations:** 1 Instituto de Síntesis Química y Catálisis Homogénea, CSIC - Universidad de Zaragoza, Pedro Cerbuna 12, 50009 Zaragoza, Spain

**Keywords:** C–H functionalization, carbonylation, glutamic acid, glutamide, palladium, phenylglycine

## Abstract

A new method for the regioselective synthesis of 2-alkoxycarbonyl- and 2-(aminocarbonyl)phenylglycinate methyl esters has been developed. The reaction of the orthopalladated complex [Pd(μ-Cl)(C_6_H_4_(CH(CO_2_Me)NMe_2_)-2)]_2_ (**1**) with nucleophiles HNu under a CO atmosphere results in the selective incorporation of the C(O)Nu moiety to the phenyl ring and formation of the carbonyl species *ortho*-C_6_H_4_(C(O)Nu)(CH(CO_2_Me)NMe_2_) (**2a**–**j**) (Nu = OR, NHR, NR_2_). Compounds **2a**–**j** are conformationally restricted analogues of glutamic acid and glutamine and are interesting due to their biological and pharmacological properties. The reaction of [Pd(μ-Cl)(C_6_H_4_(CH(CO_2_Me)NHTf)-2)]_2_ (**3**) with nucleophiles in a CO atmosphere results, however, in the formation of the cyclic isoindolinone or the open 2-carboxyphenylglycine methyl esters, with the reaction outcome being driven by the choice of the solvent.

## Introduction

The selective functionalization of organic molecules is, at the present time, one of the most developed areas of organic and organometallic chemistry. Several factors have contributed to this spectacular growth. The main one is the use of transition metals, such as Rh, Ru, Pd, Pt or Au, with the capability of activating and breaking C–H bonds and, thus, transforming the inert C–H unit into the reactive C–M group (M = transition metal) [1–3]. In addition, the introduction of the concept of a "directing group" enables the attack of the metal on a unique position [4], therefore affording highly selective processes and avoiding the obtainment of unwanted isomers.

Probably the aspect of this method of synthesis with the greatest impact is the oxidative coupling of two C–H bonds to give a new C–C bond, because it avoids the use of prefunctionalized substrates, minimizes the amount of waste generated during the reaction and, in general, allows for the reactions to occur under mild conditions and tolerates a variety of functional groups [5–10]. This is advantageous when reactive or sensitive fragments are present in the molecular scaffold.

**Scheme 1:**
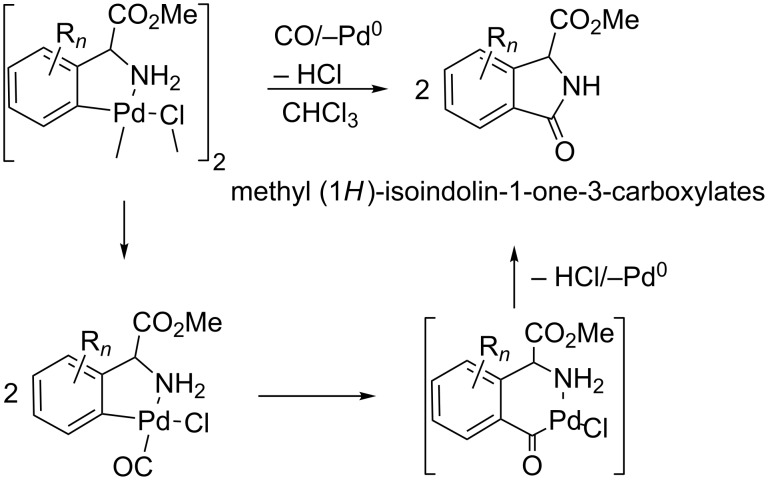
Synthesis of methyl (1*H*)-isoindolin-1-one-3-carboxylates by carbonylation of phenylglycine derivatives [12].

We are interested in the regioselective functionalization of α-amino acids [11–13], due to the extraordinary interest in these delicate molecules as building blocks of peptides and proteins, and because of their relevant biological activity. In this context, we have recently reported C–H bond activation processes on a variety of arylglycines substituted at the phenyl ring, and the corresponding synthesis of a new family of orthopalladated complexes [12]. The carbonylation of these compounds allows for a general synthesis of methyl (1*H*)-isoindolin-1-one-3-carboxylates under very mild reaction conditions, regardless of whether the substituents at the aryl ring R*_n_* are electron-withdrawing or electron-releasing. This method, shown in Scheme 1, represents a real synthetic alternative to other classical preparative pathways [12].

With the aim of expanding the scope of application of this method, we report in this paper the results obtained when other functional groups on the same starting material (methyl phenylglycinate) are changed. In particular, we have detected that the presence of different types of substituents at the nitrogen atom has a critical effect on the final outcome of the reaction and that, instead of the expected (1*H*)-isoindolin-1-ones, conformationally restricted glutamines and glutamates can be obtained. The undoubted importance of conformationally constrained aminoacids is based on the fact that their incorporation into peptides constitutes a very useful strategy to reduce their flexibility and retard enzymatic degradation. Moreover, these restricted amino acids can stabilize particular conformational features, which may lead to improvements in the biological potency if the bioactive conformation is tethered [14–16].

## Results and Discussion

### Synthesis of new orthopalladated derivatives

**Scheme 2:**
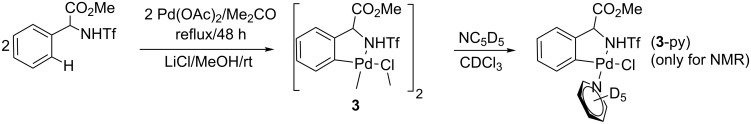
Synthesis and NMR characterization of orthometallated complex **3**.

Two phenylglycinate derivatives have been used as starting materials, one of them containing a sterically hindered N atom, protected by two methyl groups, namely [C_6_H_5_C(H)(CO_2_Me)NMe_2_] [17–18], and the other one containing a less hindered, but strongly electron-withdrawing, triflate (Tf) group [C_6_H_5_C(H)(CO_2_Me)NHTf] [19]. The orthopalladation of [C_6_H_5_C(H)(CO_2_Me)NMe_2_] has been reported previously by Ryabov and Beck [17–18], and affords complex **1** by heating of Pd(OAc)_2_ and [C_6_H_5_C(H)(CO_2_Me)NMe_2_] in acetic acid (55–60 °C over 15–20 min), followed by stirring at room temperature for 2–3 days. In this way, complex **1** is obtained in 50% yield. We did not use this method, and we present here an optimized synthesis of complex **1**, which is achieved by heating a solution of Pd(OAc)_2_ with [C_6_H_5_C(H)(CO_2_Me)NMe_2_] (1:1 molar ratio) in acetone under reflux for 24 h, followed by the typical metathesis of acetate by chloride bridging ligands in MeOH. Our improved procedure takes place in a shorter reaction time (1 versus 3 days) and affords analytically pure complex **1** in yields typically higher than 65%. The characterization of **1** was performed by comparison of its spectral data with those previously reported [18]. On the other hand, the reaction of [C_6_H_5_C(H)(CO_2_Me)NHTf] with Pd(OAc)_2_ (1:1 molar ratio) affords the orthometallated [Pd(µ-Cl)(C_6_H_4_CH(CO_2_Me)NHTf-2)]_2_ (**3**), after metathesis of acetate by chloride bridging ligands, as shown in Scheme 2. In this case the reaction also takes place in acetone under reflux, but 48 h of heating is necessary to achieve completion. Complex **3** was characterized following the usual techniques. Both microanalytical and mass spectral data are in good agreement with the proposed dinuclear stoichiometry for **3**. The ^1^H NMR spectrum of **3** shows broad signals, probably due to different equilibrium processes. These could involve the interconversion between the two possible diastereoisomers (*RR*/*SS* and *RS*/*SR*) through cleavage of the chloride bridges, as well as the potential formation of *cisoid* and *transoid* geometric isomers. The breakage of the chloride bridging system by NC_5_D_5_ and "in situ" formation of the corresponding mononuclear derivative (**3**-py, see Scheme 2), which is static on the NMR time scale, simplifies notably the NMR spectra. The ^1^H NMR spectrum shows then the presence of four well-spread signals, one of them (H6) strongly shifted upfield due to the anisotropic shielding of the *cis*-pyridine ring. This observation, together with the presence of six different peaks in the ^13^C NMR spectrum, one of them clearly deshielded (C1, 151.41 ppm), points to the presence of the PdC_6_H_4_ unit. All the other features of the NMR data are in keeping with the structure depicted in Scheme 2.

### Synthesis of conformationally restricted glutamates and glutamines

**Scheme 3:**
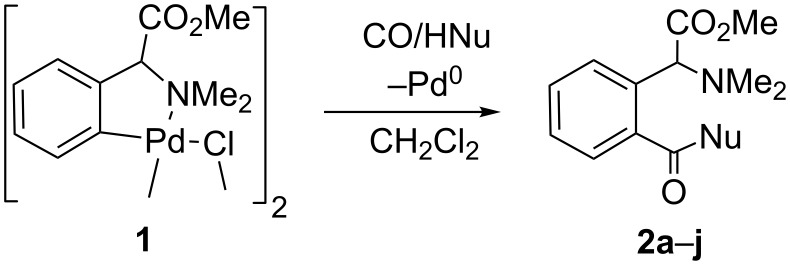
Carbonylation of **1** to afford glutamate and glutamine derivatives **2a**–**j**.

**Figure 1:**
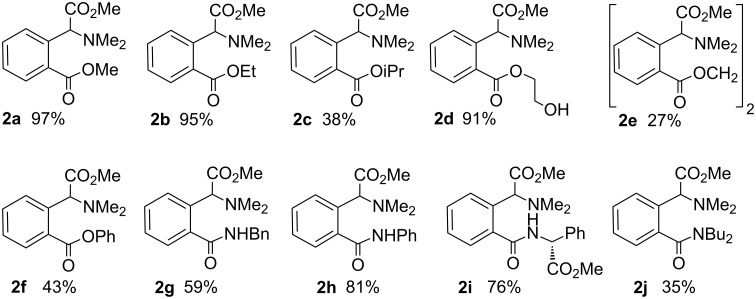
Scope of the carbonylation reaction.

Complex **1** reacts with CO in the presence of alcohols or amines (even aminoesters) affording the corresponding alkoxycarbonylated (**2a**–**f**) or aminocarbonylated species (**2g**–**j**), as shown in Scheme 3 and Figure 1, under very mild reaction conditions (CH_2_Cl_2_, 1 atm CO, 25 °C).

The clear formation of black palladium indicates the progress of the reaction, which is completed typically in 16 h in all studied cases. After removal of the Pd^0^ the workup of the reaction is very simple, since the evaporation of the solvent affords **2a**–**j** as analytically pure yellow oils. Compounds **2a**–**f** can be considered as glutamic acid derivatives, while **2g**–**j** are analogues of glutamine, in which the β- and γ-positions belong to an aryl ring and display, therefore, a severe conformational restriction.

The present method appears to be quite general, since it is valid for a wide range of alcohols and amines. In the case of alcohols, primary (**2a**, **2b**, **2d**, **2e**) and secondary (**2c**) aliphatic alcohols, and even arylic substrates (**2f**) have been incorporated into the phenylglycine scaffold. Very good yields are obtained with acidic alcohols, such as methanol (**2a**), ethanol (**2b**) or even 1,2-ethanediol (**2d**). These values drop when 2-propanol (**2c**) or phenol (**2f**) are used, and moderate yields are obtained (≈40%), whereas no reaction at all is observed for bulky tertiary alcohols, for example when Me_3_COH is used.

In alkoxycarbonylation reactions the nucleophile finally incorporated into the carbonyl group (an alkoxide) usually comes from the reaction solvent (an alcohol). This fact guarantees the full displacement of the reaction, but sometimes hampers the purification of the target products, mainly when alcohols of high boiling point and/or viscosity are involved. However, in our method, CH_2_Cl_2_ is used as the solvent and stoichiometric amounts of the nucleophiles are used instead, without any problem in the purification step.

**Scheme 4:**
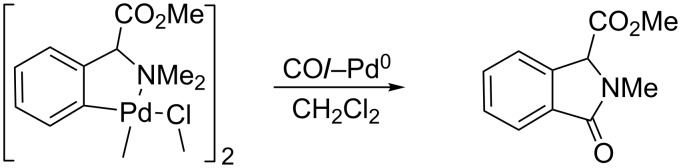
Reaction of **1** and CO in CH_2_Cl_2_ [18].

Interestingly, there is a clear difference in the reactivity of **1** with CO, depending on the presence or lack of nucleophiles. The reaction of **1** with CO in CH_2_Cl_2_ has been reported previously by Beck [18], and this process affords the γ-lactam displayed in Scheme 4. Assuming the mechanism shown in Scheme 1, it seems that, in the absence of any other nucleophile, the intramolecular C–N coupling takes place with concomitant formation of a *N,N*-dimethylisoindolinonium salt, which undergoes further elimination of a methyl group by 1,2-shift of the Me unit from the N atom to the Pd centre, as previously reported by Heck et al. [20].

As we have shown previously in Scheme 3, in the presence of nucleophiles the process results in the formation of conformationally restricted glutamate derivatives. This is mainly due to the fact that the demethylation of the NMe_2_ unit shown in Scheme 4 is not a very favourable process, and the reaction can take a different outcome, especially if alternative pathways are accessible. Taking into account these facts, we can propose a sensible explanation for the different reactivity. Therefore, the attack of the oxygen of an O-bonded alcohol on the electrophilic acyl carbon in our complexes seems to be favoured, since no demethylation is involved, and the C–O coupling occurs selectively instead of the intramolecular C–N bond formation. It seems that the reaction is driven by the pathway that tends to avoid the demethylation, while the comparison of the different nucleophilic abilities of the species coordinated to the metal (O-bonded alcohols versus N-bonded amines) plays in this case only a minor role.

Using the same arguments we can explain the different reactivity found for **1**, and shown in Scheme 3, when compared to related Pd complexes previously reported by us [12], resumed in Scheme 1. Therefore, the synthesis of the methyl (1*H*)-isoindolin-1-one-3-carboxylates by carbonylation of [Pd(μ-Cl)(C_6_H_4_CH(CO_2_Me)NH_2_-2)]_2_ occurs by C–N coupling, irrespective of the presence of additional nucleophiles, since the cyclization generates an isoindolinonium salt, from which it is relatively easy to promote a simple deprotonation.

Very interestingly, the reactivity of **1** is not limited to the addition of alcohols, and primary amines, secondary amines, and even α-aminoesters can also be coupled to the *N,N*-dimethylarylglycine fragment, as stated above. Then, the reaction of **1** with CO, in CH_2_Cl_2_, and in the presence of stoichiometric amounts of benzylamine, aniline, methyl (*R*)-phenylglycinate or di-*n*-butylamine, occurs with smooth insertion of CO into the Pd-C_aryl_ bond and further incorporation of the C(O)NHCH_2_Ph (**2g**), C(O)NHPh (**2h**), C(O)NHCH(CO_2_Me)Ph (**2i**) or C(O)NBu_2_ (**2j**) moieties into the *ortho*-position of the C_6_H_4_C(H)(CO_2_Me)NMe_2_ ligand. This results in the synthesis of the corresponding conformationally restricted glutamines **2g**–**j** in moderate to good yields, as shown in Figure 1. This means that the process can be efficiently performed not only with a variety of O-nucleophiles, but also with different types of N-nucleophiles.

In comparison with other aminocarbonylations found in the recent literature [21–24], our method is remarkable since it occurs under very mild reaction conditions (1 atm CO, 25 °C) and, mainly, because it occurs through C–H bond activation processes without the need to use prefunctionalized substrates. Typical aminocarbonylations catalysed by Pd usually start from the corresponding iodides or bromides, and require high CO pressures and high reaction temperatures. Obviously, further efforts in our systems have to be directed to the transformation of the stoichiometric process into a catalytic one, a challenge that is still not accomplished in the case of the aminocarbonylation, even though several catalytic examples are known of the related alkoxycarbonylation reaction [25–30].

**Scheme 5:**
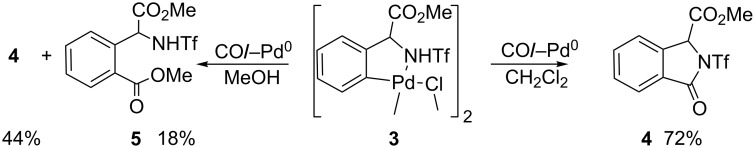
Reactivity of **3** with CO in the presence (left) and absence (right) of nucleophiles.

Once we had determined the reactivity of complex **1**, having a *N,N*-dimethyl-phenylglycine ligand, we focused our attention on complex **3**, possessing a triflate as a *N*-protecting group, in order to study the influence of the substituents at the N atom in the carbonylation further. The reaction of **3** with CO (1 atm) in CH_2_Cl_2_ at room temperature, that is, in the absence of nucleophiles, occurs with C–N coupling and formation of the methyl 3-oxo-2-((trifluoromethyl)sulfonyl)isoindoline-1-carboxylate (**4**) in good yields, as shown in Scheme 5 (right).

This means that the N atom is still nucleophilic enough to promote the cyclization, in spite of the presence of the highly electron-withdrawing triflate group. It is also clear that, after C–N coupling, the resulting ammonium salt eliminates easily HCl (formally) affording the neutral amine, in close similarity to the process shown in Scheme 1. However, when the reaction of **3** with CO (1 atm) is performed in MeOH, a mixture of the compounds derived from intramolecular cyclization (**4**) and alkoxycarbonylation (**5**) is obtained (molar ratio **4**/**5** 2.4:1). This mixture can be separated by column chromatography, and pure isolated compound **5** has been characterized as containing the NHTf group and two different CO_2_Me moieties, as represented in Scheme 5 (left). This result can be interpreted as a competing reaction between the nucleophilic abilities of the N atom of the glycine moiety and the oxygen atom of the methanol, which in this case is the reaction solvent. It is clear that the introduction of the triflate group decreases the electron density of the N atom, which is now less nucleophilic in comparison, for example, with the complex containing the NH_2_ unit, shown in Scheme 1. In that case the N was quite nucleophilic, and cyclization occurred regardless of the solvent used for the reaction [12]. In the present case the N is less nucleophilic and competes with other nucleophiles, giving mixtures **4** and **5** in the presence of methanol. Obviously, in absence of additional nucleophiles, **4** is obtained selectively. We attempted several reaction conditions in order to prepare selectively compound **5**, but it seems to be difficult to quench the intramolecular cyclization, and in all studied cases the **4**/**5** mixture is obtained. Due to this fact, we have not studied other alcohols.

## Conclusion

The reactivity of the orthopalladated dimers [Pd(µ-Cl){C_6_H_4_(CH(CO_2_Me)NR_2_)-2}]_2_ (NR_2_ = NMe_2_, NHTf) towards CO in the presence of alcohols or amines as nucleophiles allows for the synthesis of conformationally restricted glutamates or glutamines, respectively, through alkoxycarbonylation or aminocarbonylation intermolecular processes. In spite of the presence of an intramolecular nucleophile (the N atom of the NR_2_ group), the formation of the cyclic isoindolinone derivatives has been observed only in one case. This means that the nitrogen atoms of the NMe_2_ or the NHTf groups behave as weaker nucleophiles than the oxygen or nitrogen atoms of the external nucleophiles involved (alcohols, amines). In addition, the results also show that the nucleophilic abilities of the N atom in the starting materials [Pd(µ-Cl)(C_6_H_4_CH(CO_2_Me)NR_2_)]_2_ (NR_2_ = NMe_2_, NHTf) are weaker than those observed in [Pd(µ-Cl)(C_6_H_4_CH(CO_2_Me)NH_2_)]_2_, for which a systematic intramolecular aminocarbonylation was observed.

## Experimental

**General Methods.** The general methods are reported in the Supporting Information File 1. The complex [Pd(μ-Cl)(C_6_H_4_CH(CO_2_Me)NMe_2_-2)]_2_ (**1**) has been prepared following previously reported procedures [17–18].

### Synthesis of methyl *N,N*-dimethyl-α-(2-methoxycarbonylphenyl)glycinate (**2a**)

Methanol (13 μL, 0.300 mmol) was added to a solution of **1** (100.0 mg, 0.150 mmol) in CH_2_Cl_2_ (10 mL), and the resulting mixture was stirred under a CO atmosphere for 16 h. Decomposition to black metallic palladium was observed. The mixture was filtered through a plug of Celite. The light yellow solution was washed with water (3 × 20 mL), dried over MgSO_4_, filtered and evaporated to give compound **2a** as a yellow oil. Yield: 72.9 mg, 0.290 mmol, 97%.

^1^H NMR (300 MHz, CDCl_3_) δ 7.83 (d, *J* = 7.7 Hz, 1H, C_6_H_4_), 7.68 (d, *J* = 7.7 Hz, 1H, C_6_H_4_), 7.50 (t, *J* = 7.7 Hz, 1H, C_6_H_4_), 7.35 (t, *J* = 7.7 Hz, 1H, C_6_H_4_), 5.12 (s, 1H, CH), 3.89 (s, 3H, OMe), 3.69 (s, 3H, OMe), 2.31 (s, 6H, NMe_2_); ^13^C NMR (75 MHz, CDCl_3_) δ 172.08 (s, CO), 168.36 (s, CO), 137.60 (s, C), 131.96 (s, CH), 130.99 (s, C), 130.32 (s, CH), 129.03 (s, CH), 127.88 (s, CH), 68.42 (s, CH), 52.34 (s, OCH_3_), 51.88 (s, OCH_3_), 42.97 (s, NMe_2_); IR (ν, cm^−1^) 1724 (C=O), 1257 (C-O); ESIMS (positive mode) (*m*/*z*): 251.9 [M + H]^+^; anal. calcd for C_13_H_17_NO_4_ (251.12): C, 62.14; H, 6.82; N, 5.57; found: C, 62.35; H, 6.91; N, 5.36.

### Synthesis of [Pd(μ-Cl)(C_6_H_4_CH(CO_2_Me)NHTf-2)]_2_ (**3**)

To a solution of Pd(OAc)_2_ (421.1 mg, 1.836 mmol) in acetone (30 mL), PhCH(CO_2_Me)NHTf [19] (545.8 mg, 1.836 mmol) was added, and the resulting mixture was heated under reflux for 48 h. After the reaction time, the solution was evaporated to dryness, the residue was treated with CH_2_Cl_2_ (40 mL), and the resulting suspension was filtered over a Celite pad. The resulting clear solution was again evaporated to dryness, and the residue was dissolved in MeOH and allowed to react with NaCl (243.6 mg, 4.167 mmol) at room temperature for 4 h. The pale yellow solution was evaporated to dryness, the dry residue extracted with CH_2_Cl_2_ (30 mL), and the resulting suspension filtered to eliminate the excess of NaCl. Evaporation of this clear solution and treatment of the residue with pentane afforded **3** as a yellow–brownish solid. Yield: 452.6 mg, 0.516 mmol, 56.2% yield.

^1^H NMR (300 MHz, CDCl_3_ + py-*d*_5_) δ 7.21 (dd, *J* = 7.6, 1.5 Hz, 1H, C_6_H_4_), 6.94 (td, *J* = 7.4, 1.2 Hz, 1H, C_6_H_4_), 6.71 (td, *J* = 7.5, 1.5 Hz, 1H, C_6_H_4_), 5.91 (dd, *J* = 7.7, 1.2 Hz, 1H, C_6_H_4_), 5.38 (s, 1H, CH), 3.78 (s, 3H, OCH_3_), 2.24 (s, 1H, NH); ^13^C NMR (75 MHz, CDCl_3_+py-*d*_5_) δ 173.94 (s, CO), 151.41 (s, C, C_6_H_4_), 149.54 (m, CD, py), 146.10 (s, C, C_6_H_4_), 135.55 (m, CD, py), 132.49 (s, CH, C_6_H_4_), 125.57 (s, CH, C_6_H_4_), 124.46 (s, CH, C_6_H_4_), 123.29 (m, CD, py), 122.69 (s, CH, C_6_H_4_), 120.82 (q, *J* = 324.6 Hz, CF_3_), 72.37 (s, CH), 52.30 (s, OCH_3_); ^19^F NMR (282 MHz, CDCl_3_ + py-*d*_5_) δ −76.71 (s, CF_3_); IR (ν, cm^−1^): 3375 (br, N-H), 1742 (νCOO); ESIMS (positive mode) (*m*/*z*): 439 [M/2 + H]^+^; anal. calcd for C_20_H_18_Cl_2_F_6_N_2_O_8_Pd_2_S_2_ (876.23): C, 27.41; H, 2.07; N, 3.20; S, 7.32; found: C, 26.93; H, 2.02; N, 3.45; S, 6.98.

### Synthesis of methyl 3-oxo-2-((trifluoromethyl)sulfonyl)isoindoline-1-carboxylate (**4**)

A solution of **3** (50.0 mg, 0.057 mmol) in dichloromethane was stirred under a CO atmosphere for 16 h. Decomposition to black palladium was observed. The mixture was filtered through a plug of Celite, and the yellow solution was washed with water (3 × 20 mL), dried over MgSO_4_, filtered and evaporated to give **4** as a yellow oil. Yield: 26.7 mg, 0.083 mmol, 72%.

^1^H NMR (300 MHz, CDCl_3_) δ 7.98 (dt, *J* = 7.7, 1.0 Hz, 1H, C_6_H_4_), 7.79 (td, *J* = 7.6, 1.2 Hz, 1H, C_6_H_4_), 7.68 (dd, *J* = 7.8, 0.9 Hz, 1H, C_6_H_4_), 7.65 (td, *J* = 7.5, 0.7 Hz, 1H, C_6_H_4_), 5.72 (s, 1H, CH), 3.85 (s, 3H, OCH_3_); ^13^C NMR (75 MHz, CDCl_3_) δ 166.65 (s, CO), 164.37 (s, CO), 139.43 (s, C), 135.84 (s, CH), 130.87 (s, CH), 127.63 (s, C), 126.49 (s, CH), 123.54 (s, CH), 119.59 (q, *J* = 323.5 Hz, CF_3_), 63.20 (s, CH), 53.94 (s, OCH_3_); ^19^F NMR (282 MHz, CDCl_3_) δ −74.04 (s, CF_3_); IR (ν, cm^−1^): 1758 (COO). ESIMS (positive mode) (*m*/*z*): 324.0 [M + H]^+^; anal. calcd for C_11_H_8_F_3_NO_5_S (323.01): C, 40.87; H, 2.49; N, 4.33; S, 9.92; found: C, 40.94; H, 2.53; N, 4.41; S, 10.05.

### Synthesis of methyl *N*-trifluoromethylsulfonamido-α-(2-methoxycarbonylphenyl)glycinate (**5**)

A solution of **3** (100.0 mg, 0.114 mmol) in methanol was stirred under a CO atmosphere for 16 h. During the reaction, the formation of Pd^0^ was evident. The black material was eliminated by filtration through a plug of Celite, and the resulting light yellow solution was washed with water (3 × 20 mL), dried over MgSO_4_, filtered and evaporated to give an oily residue characterized as the mixture of compounds **4** and **5**. This mixture was separated by column chromatography (silica, hexane/CH_2_Cl_2_: 3/7), yielding pure **5** as a colourless oil. Yield: 14.2 mg, 0.040 mmol, 18%.

^1^H NMR (300 MHz, CDCl_3_) δ 8.10 (dd, *J* = 7.8, 1.5 Hz, 1H, C_6_H_4_), 7.61 (td, *J* = 7.5, 1.5 Hz, 1H, C_6_H_4_), 7.50 (td, *J* = 7.6, 1.4 Hz, 1H, C_6_H_4_), 7.41 (dd, *J* = 7.6, 1.5 Hz, 1H, C_6_H_4_), 7.00 (d, *J* = 9.4 Hz, 1H, NH), 5.42 (d, *J* = 9.3 Hz, 1H, CH), 3.92 (s, 3H, OCH_3_), 3.75 (s, 3H, OCH_3_); ^13^C NMR (75 MHz, CDCl_3_) δ 168.89 (s, CO), 168.43 (s, CO), 137.53 (s, C), 133.86 (s, C), 132.59 (s, CH), 132.01 (s, CH), 129.68 (s, CH), 127.30 (s, CH), 119.53 (q, *J* = 320.7 Hz, CF_3_), 61.35 (s, CH), 53.42 (s, OCH_3_), 53.03 (s, OCH_3_); ^19^F NMR (282 MHz, CDCl_3_) δ −77.53 (s, CF_3_); IR (ν, cm^−1^): 3282 (br, N-H), 1747 (COO), 1711 (COO); ESIMS (positive mode) (*m*/*z*): 324.2 [M − OMe]^+^, 356.0 [M + H]^+^; anal. calcd for C_12_H_12_F_3_NO_6_S (355.03): C, 40.57; H, 3.40; N, 3.94; S, 9.03; found: C, 40.42; H, 3.24; N, 3.82; S, 8.93.

## Supporting Information

File 1 General methods and experimental and analytical data of compounds **2b**–**j**.
