# Supplementary material for: Synthesis of conformationally restricted glutamate and glutamine derivatives from carbonylation of orthopalladated phenylglycine derivatives
Source: Beilstein J Org Chem. 2012 Sep 18;8:1569–75. doi: 10.3762/bjoc.8.179 (PMC3510988; doi:10.3762/bjoc.8.179)
Supplement: File 1 — General methods and experimental and analytical data of compounds 2b–j. [file Beilstein_J_Org_Chem-08-1569-s001.pdf]

Supporting Information

for

Synthesis of conformationally restricted glutamate and glutamine derivatives from carbonylation of orthopalladated phenylglycine derivatives

Esteban P. Urriolabeitia*, Eduardo Laga, and Carlos Cativiela

Address: Instituto de Síntesis Química y Catálisis Homogénea, CSIC - Universidad de Zaragoza, Pedro Cerbuna 12, 50009 Zaragoza, Spain

Email: Esteban P. Urriolabeitia - esteban@unizar.es

* Corresponding author

General methods and experimental and analytical data of compounds 2b–j

General Methods. Elemental analyses were carried out on a Perkin-Elmer 2400-B microanalyzer. Infrared spectra (4000–200 cm^{-1}) were recorded on a Perkin-Elmer Spectrum One from nujol mulls between polyethylene sheets. The ^1H, $^{13}C\{^1H\}$ and ^{19}F NMR spectra were recorded in CD_2Cl_2, $CDCl_3$ or acetone-d_6 solutions at 25 °C on Bruker Avance-300 and Avance-400 spectrometers (δ, ppm; *J*, Hz); ^1H and $^{13}C\{^1H\}$ spectra were referenced by using the solvent signal as an internal standard, while ^{19}F NMR spectra were referenced to $CFCl_3$. Electrospray Ionization (ESI)/Atmospheric Pressure Chemical Ionization (APCI) mass spectra were recorded on an Esquire 3000 ion-trap mass spectrometer (Bruker Daltonic GmbH, Bremen, Germany) equipped with a standard ESI/APCI source. Samples were introduced by direct infusion with a syringe pump. Nitrogen served both as the nebulizer gas and the dry gas. Helium served as a cooling gas for the ion trap and collision gas for MSn experiments. Other MS (MALDI-DIT) were recorded from CH_2Cl_2 solutions on a Bruker MicroFlex spectrometer.

Synthesis of methyl *N,N*-dimethyl-α-(2-methoxycarbonylphenyl)glycinate (2a)

Methanol (13 µL, 0.300 mmol) was added to a solution of **1** (100.0 mg, 0.150 mmol) in CH_2Cl_2 (10 mL), and the resulting mixture was stirred under a CO atmosphere for 16 h. Decomposition to black metallic palladium was observed. The mixture was filtered through a plug of Celite. The light yellow solution was washed with water (3 × 20 mL), dried over $MgSO_4$, filtered and evaporated to give compound **2a** as a yellow oil. Yield: 72.9 mg, 0.290 mmol, 97%.

^1H NMR (300 MHz, $CDCl_3$) δ 7.83 (d, *J* = 7.7 Hz, 1H, C_6H_4), 7.68 (d, *J* = 7.7 Hz, 1H, C_6H_4), 7.50 (t, *J* = 7.7 Hz, 1H, C_6H_4), 7.35 (t, *J* = 7.7 Hz, 1H, C_6H_4), 5.12 (s, 1H, CH), 3.89 (s, 3H, OMe), 3.69 (s, 3H, OMe), 2.31 (s, 6H, NMe_2); ^{13}C NMR (75 MHz, $CDCl_3$) δ 172.08 (s, CO), 168.36 (s, CO), 137.60 (s, C), 131.96 (s, CH), 130.99 (s, C),

130.32 (s, CH), 129.03 (s, CH), 127.88 (s, CH), 68.42 (s, CH), 52.34 (s, OCH_3), 51.88 (s, OCH_3), 42.97 (s, NMe_2); IR (ν, cm^{-1}): 1724 (C=O), 1257 (C-O); ESIMS (positive mode) (*m*/*z*): 251.9 $[M + H]^+$; anal. Calcd for $C_{13}H_{17}NO_4$ (251.12): C, 62.14; H, 6.82; N, 5.57; found: C, 62.35; H, 6.91; N, 5.36.

Synthesis of methyl *N,N*-dimethyl-α-(2-ethoxycarbonylphenyl)glycinate (2b)

Compound **2b** was prepared following the same experimental procedure to that described for **2a**. Therefore, ethanol (18 μL, 0.300 mmol) was reacted with **1** (100.0 mg, 0.150 mmol) in CH_2Cl_2 (10 mL) under a CO atmosphere for 16 h to give compound **2b** as a yellow oil. Yield: 75.9 mg, 0.286 mmol, 95%.

^1H NMR (300 MHz, $CDCl_3$) δ 7.82 (dd, *J* = 7.8, 1.4 Hz, 1H, C_6H_4), 7.68 (dd, *J* = 8.0, 1.4 Hz, 1H, C_6H_4), 7.49 (td, *J* = 7.6, 1.5 Hz, 1H, C_6H_4), 7.35 (td, *J* = 7.5, 1.3 Hz, 1H, C_6H_4), 5.12 (s, 1H, CH), 4.37 (q, *J* = 7.1 Hz, 2H, OCH_2), 3.69 (s, 3H, OCH_3), 2.31 (s, 6H, NMe_2), 1.40 (t, *J* = 7.1 Hz, 3H, CH_3); ^{13}C NMR (75 MHz, $CDCl_3$) δ 172.12 (s, CO), 168.03 (s, CO), 137.45 (s, C), 131.75 (s, CH), 131.63 (s, C), 130.18 (s, CH), 129.04 (s, CH), 127.86 (s, CH), 68.51 (s, CH), 61.33 (s, OCH_2), 51.83 (s, OCH_3), 43.00 (s, NMe_2), 14.41 (s, CH_3); IR (ν, cm^{-1}): 1739 (C=O), 1713 (C=O), 1257 (C-O); ESIMS (positive mode) (*m*/*z*): 266.1 $[M + H]^+$; anal. Calcd for $C_{14}H_{19}NO_4$ (265.13): C, 63.38; H, 7.22; N, 5.28; found: C, 63.24; H, 7.14; N, 5.36.

Synthesis of methyl *N,N*-dimethyl-α-(2-isopropoxycarbonylphenyl)glycinate (2c)

Compound **2c** was prepared following the same experimental procedure to that described for **2a**. Therefore, 2-propanol (23 μL, 0.300 mmol) was reacted with **1** (100.0 mg, 0.150 mmol) in CH_2Cl_2 (10 mL) under a CO atmosphere for 16 h to give compound **2c** as a yellow oil. Yield: 31.6 mg, 0.113 mmol, 38%.

^1H NMR (300 MHz, $CDCl_3$) δ 7.76 (dd, *J* = 7.7, 1.6 Hz, 1H, C_6H_4), 7.67 (dd, *J* = 7.9, 1.3 Hz, 1H, C_6H_4), 7.47 (td, *J* = 7.6, 1.5 Hz, 1H, C_6H_4), 7.33 (td, *J* = 7.6, 1.4 Hz, 1H, C_6H_4), 5.24 (hept, *J* = 6.3 Hz, 1H, OCH), 5.09 (s, 1H, CH), 3.68 (s, 3H, OCH_3), 2.29 (s, 6H, NMe_2), 1.38 (d, *J* = 6.3 Hz, 3H, CH_3), 1.37 (d, *J* = 6.3 Hz, 3H, CH_3); ^{13}C NMR (75 MHz, $CDCl_3$) δ 172.15 (s, CO), 167.58 (s, CO), 137.15 (s, C), 132.16 (s, C), 131.56 (s, CH), 129.96 (s, CH), 128.98 (s, CH), 127.82 (s, CH), 68.87 (s, CH), 68.47 (s, CH), 51.82 (s, OCH_3), 43.05 (s, NMe_2), 22.0(s, CH_3), 21.99(s, CH_3); IR (ν, cm^{-1}): 1735 (C=O), 1713 (C=O), 1258 (C-O); ESIMS (positive mode) (*m*/*z*): 280.1 $[M + H]^+$; anal. Calcd for $C_{15}H_{21}NO_4$ (279.15): C, 64.50; H, 7.58; N, 5.01; found: C, 64.61; H, 7.55; N, 4.97.

Synthesis of methyl *N,N*-dimethyl-α-[2-(2-hydroxy)ethoxycarbonylphenyl] glycinate (2d)

Compound **2d** was prepared following the same experimental procedure to that described for **2a**. Therefore, 1,2-ethanediol (17 μL, 0.300 mmol) was reacted with **1** (100.0 mg, 0.150 mmol) in CH_2Cl_2 (10 mL) under a CO atmosphere for 16 h to give compound **2d** as a yellow oil. Yield: 53.7 mg, 0.191 mmol, 91%.

^1H NMR (300 MHz, $CDCl_3$) δ 7.80 (dd, *J* = 7.8, 1.4 Hz, 1H, C_6H_4), 7.71 (dd, *J* = 7.9, 1.3 Hz, 1H, C_6H_4), 7.50 (td, *J* = 7.6, 1.5 Hz, 1H, C_6H_4), 7.37 (td, *J* = 7.5, 1.3 Hz, 1H, C_6H_4), 4.91 (s, 1H, CH), 4.43 (m, 2H, OCH_2), 3.92 (m, 2H, OCH_2), 3.70 (s, 3H, OCH_3), 3.21 (s br, 1H, OH), 2.29 (s, 6H, NMe_2); ^{13}C NMR (75 MHz, $CDCl_3$) δ 171.99 (s, CO), 168.27 (s, CO), 136.44 (s, C), 131.94 (s, CH), 131.52 (s, C), 130.27 (s, CH), 129.51 (s, CH), 128.19 (s, CH), 69.93 (s, CH), 67.31 (s, OCH_2), 60.89 (s, OCH_2), 52.19 (s, OCH_3), 43.21 (s, NMe_2); IR (ν, cm^{-1}): 3424 (O-H), 1716 (br, C=O), 1252 (C-O); ESIMS (positive mode) (*m*/*z*): 282.1 $[M + H]^+$; anal. Calcd for $C_{14}H_{19}NO_5$ (281.13): C, 59.78; H, 6.81; N, 4.98; found: C, 58.57; H, 6.71; N, 4.99.

Synthesis of ethane-1,2-diyl bis(2-(1-(dimethylamino)-2-methoxy-2-oxoethyl)benzoate) (2e)

Compound **2e** was prepared following the same experimental procedure to that described for **2a**. Therefore, 1,2-ethanediol (8 μL, 0.150 mmol) was reacted with **1** (100.0 mg, 0.150 mmol) in CH_2Cl_2 (10 mL) under a CO atmosphere for 16 h to give compound **2e** as a yellow oil. Yield: 20.1 mg, 0.040 mmol, 27%.

^1H NMR (300 MHz, $CDCl_3$) δ 7.86 (d, *J* = 7.5 Hz, 1H, C_6H_4), 7.68 (d, *J* = 7.9 Hz, 1H, C_6H_4), 7.51 (d, *J* = 7.5 Hz, 1H, C_6H_4), 7.34 (td, *J* = 7.5, 1H, C_6H_4), 5.13 (s, 1H, CH), 4.64 (s, 2H, OCH_2), 3.67 (s, 3H, OCH_3), 2.28 (s, 6H, NMe_2); ^{13}C NMR (75 MHz, $CDCl_3$) δ 171.96 (s, CO), 167.63 (s, CO), 137.92 (s, C), 132.14 (s, CH), 130.73 (s, C), 130.43 (s, CH), 129.14 (s, CH), 127.93 (s, CH), 68.44 (s, CH), 63.00 (s, OCH_2), 51.82 (s, OCH_3), 42.89 (s, NMe_2); IR (v, cm^{-1}): 1723 (br, C=O), 1256 (C-O); ESIMS (positive mode) (*m*/*z*): 501.2 $[M + H]^+$; anal. Calcd for $C_{26}H_{32}N_2O_8$ (500.22): C, 62.39; H, 6.44; N, 5.60; found: C, 62.24; H, 6.52; N, 5.64.

Synthesis of methyl *N,N*-dimethyl-α-(2-phenoxycarbonylphenyl)glycinate (2f)

Compound **2f** was prepared following the same experimental procedure to that described for **2a**. Therefore, phenol (28.2 mg, 0.300 mmol) was reacted with **1** (100.0 mg, 0.150 mmol) in CH_2Cl_2 (10 mL) under a CO atmosphere for 16 h to give compound **2f** as a yellow oil. Yield: 40.3 mg, 0.129 mmol, 43%.

^1H NMR (300 MHz, $CDCl_3$) δ 8.11 (d, *J* = 7.7 Hz, 1H, C_6H_4), 7.79 (d, *J* = 7.9 Hz, 1H, C_6H_4), 7.62 (t, *J* = 7.5 Hz, 1H, C_6H_4), 7.49 (t, *J* = 7.9 Hz, 2H, C_6H_5), 7.34 (t, *J* = 7.5 Hz, 1H, C_6H_4), 7.27 (m, 2H, C_6H_5), 6.87 (m, 1H, C_6H_5), 5.31 (s, 1H, CH), 3.76 (s, 3H, OCH_3), 2.41 (s, 6H, NMe_2); ^{13}C NMR (75 MHz, $CDCl_3$) δ 172.03 (s, CO), 166.47 (s, CO), 150.91 (s, C), 138.20 (s, C), 132.44 (s, CH), 130.67 (s, CH), 130.54 (s, C), 129.65 (s, CH), 129.21 (s, CH), 128.07 (s, CH), 126.12 (s, CH), 121.79 (s, CH),

68.32 (s, CH), 51.88 (s, OCH_3), 42.88 (s, NMe_2); IR (ν, cm^{-1}): 1735 (br, C=O), 1257 (C-O); ESIMS (positive mode) (*m*/*z*): 314.2 $[M + H]^+$; anal. Calcd for $C_{18}H_{19}NO_4$ (313.13): C, 68.99; H, 6.11; N, 4.47; found: C, 69.02; H, 6.03; N, 4.51.

Synthesis of methyl *N,N*-dimethyl-α-(2-benzylaminocarbonylphenyl)glycinate (2g)

Benzylamine (33 μL, 0.300 mmol) was added to a solution of **1** (100.0 mg, 0.150 mmol) in CH_2Cl_2 (10 mL), and the resulting mixture was stirred under a CO atmosphere for 16 h. Decomposition to black metallic palladium was observed. The mixture was filtered through a plug of Celite. The light yellow solution was washed with water (3 × 20mL), dried over $MgSO_4$, filtered and evaporated to give compound **2g** as a yellow oil. Yield: 57.4 mg, 0.176 mmol, 59%.

^{1}H NMR (300 MHz, $CDCl_3$) δ 9.35 (s br, 1H, NH), 7.90 (m, 1H, C_6H_4), 7.44–7.25 (m, 8H, C_6H_4+C_6H_5), 4.71 (dd, *J* = 14.5, 5.7 Hz, 1H, CH_2), 4.57 (s, 1H, CH), 4.52 (dd, *J* = 14.5, 4.8 Hz, 1H, CH_2), 3.67 (s, 3H, OCH_3), 2.17 (s, 6H, NMe_2); ^{13}C NMR (75 MHz, $CDCl_3$) δ 171.18 (s, CO), 168.16 (s, CO), 138.58 (s, C), 137.58 (s, C), 132.65 (s, C), 130.83 (s, CH), 130.20 (s, CH), 128.84 (s, 2CH, overlaped signals), 128.79 (s, CH), 128.31 (s, CH), 127.61 (s, CH), 68.07 (s, CH), 51.72 (s, OCH_3), 44.62 (s, NCH_2), 40.31 (s, NMe_2); IR (ν, cm^{-1}): 3285 (br, N-H), 1735 (COO), 1643 (CON); ESIMS (positive mode) (*m*/*z*): 327.2 $[M + H]^+$; anal. Calcd for $C_{19}H_{22}N_2O_3$ (326.16): C, 69.92; H, 6.79; N, 8.58; found: C, 69.85; H, 6.68; N, 8.54.

Synthesis of methyl *N,N*-dimethyl-α-(2-anilinocarbonylphenyl)glycinate (2h)

Compound **2h** was prepared following the same experimental procedure to that described for **2g**. Therefore, aniline (27 μL, 0.300 mmol) was reacted with **1** (100.0 mg, 0.150 mmol) in CH_2Cl_2 (10 mL) under a CO atmosphere for 16 h to give compound **2h** as a yellow oil. Yield: 76.0 mg, 0.243 mmol, 81%.

^{1}H NMR (300 MHz, $CDCl_3$) δ 11.22 (s br, 1H, NH), 7.97 (m, 1H, C_6H_4), 7.73–7.66 (m, 2H, C_6H_5), 7.51–7.41 (m, 2H, C_6H_4), 7.40–7.32 (m, 2H, C_6H_5), 7.30 (m, 1H, C_6H_4), 7.12 (m, 1H, C_6H_5), 4.77 (s, 1H, CH), 3.71 (s, 3H, OCH_3), 2.54 (s, 6H, NMe_2);
^{13}C NMR (75 MHz, $CDCl_3$) δ 170.91 (s, CO), 166.43 (s, CO), 139.11 (s, C), 138.32 (s, C), 132.09 (s, C), 131.51 (s, CH), 130.54 (s, CH), 129.17 (s, CH), 129.13 (s, CH), 128.92 (s, CH), 124.18 (s, CH), 119.99 (s, CH), 68.25 (s, CH), 51.86 (s, OCH_3), 40.94 (s, NMe_2); IR (ν, cm^{-1}): 3286 (br, N-H), 1736 (COO), 1667 (CON); ESIMS (positive mode) (*m*/*z*): 313.2 $[M + H]^+$; anal. Calcd for $C_{18}H_{20}N_2O_3$ (312.15): C, 69.21; H, 6.45; N, 8.97; found: C, 69.35; H, 6.69; N, 9.04.

Synthesis of methyl *N,N*-dimethyl-α-[2-(α-methoxycarbonyl)benzylaminocarbonylphenyl]glycinate (2i)

Compound **2i** was prepared following the same experimental procedure to that described for **2g**. Therefore, (*R*)-phenylglycine methyl ester (49.5 mg, 0.300 mmol) was reacted with **1** (100.0 mg, 0.150 mmol) in CH_2Cl_2 (10 mL) under a CO atmosphere for 16 h to give compound **2i** as a yellow oil. Yield: 87.0 mg, 0.226 mmol, 76%. Compound **2i** was characterized as a mixture of diastereoisomers (1:0.25) by NMR methods.

NMR data. Major isomer: ^{1}H NMR (300 MHz, $CDCl_3$) δ 10.16 (d, *J* = 7.4 Hz, NH), 7.87 (m, C_6H_4), 7.48–7.22 (m, C_6H_4+C_6H_5), 5.84 (d, *J* = 7.4 Hz, NHC<u>H</u>), 4.96 (s, CH), 3.79 (s, OCH_3), 3.67 (s, OCH_3), 2.32 (s, 6H, NMe_2); $^{13}C\{^{1}H\}$ NMR (75 MHz, $CDCl_3$) δ 172.04 (s, CO), 171.19 (s, CO), 168.17 (s, CO), 137.16 (s, C), 136.11 (s, C), 132.84 (s, C), 130.94 (s, CH), 130.45 (s, CH), 129.02 (s, CH), 128.92 (s, CH), 128.80 (s, CH), 128.60 (s, CH), 127.63 (s, CH), 67.63 (s, CH), 57.59 (s, NH<u>C</u>H), 52.72 (s, OCH_3), 51.68 (s, OCH_3), 40.53 (s, NMe_2). *Minor isomer*: ^{1}H NMR (300 MHz, $CDCl_3$) δ 10.00 (d, *J* = 6.2 Hz, NH), 7.88 (m, C_6H_4), 7.48–7.22 (m, 8H, C_6H_4+C_6H_5), 5.69 (d, *J*

= 6.1 Hz, NHC$\underline{H}$), 4.58 (s, CH), 3.75 (s, OCH_3), 3.65 (s, OCH_3), 2.10 (s, NMe_2). $^{13}C\{^1H\}$ NMR (75 MHz, $CDCl_3$) δ 172.04 (s, CO), 171.33 (s, CO), 167.93 (s, CO), 136.89 (s, C), 136.55 (s, C), 132.77 (s, C), 131.19 (s, CH), 130.50 (s, CH), 129.20 (s, CH), 128.87 (s, CH), 128.85 (s, CH), 128.64 (s, CH), 127.93 (s, CH), 67.90 (s, CH), 58.05 (s, NH$\underline{C}$H), 52.67 (s, OCH_3), 51.68 (s, OCH_3), 40.13 (s, NMe_2); IR (ν, cm^{-1}): 3407 (br, N-H), 1738 (COO), 1651 (CON); ESIMS (positive mode) (*m*/*z*): 385.0 $[M + H]^+$; anal. Calcd for $C_{21}H_{24}N_2O_5$ (384.17): C, 65.61; H, 6.29; N, 7.29; found: C, 65.74; H, 6.32; N, 7.26.

Synthesis of methyl *N,N*-dimethyl-α-(2-*N*,*N*-dibutylaminocarbonylphenyl)glycinate (2j)

Compound **2i** was prepared following the same experimental procedure to that described for **2g**. Therefore, H^nBu_2 (50.4 µL, 0.300 mmol) was reacted with **1** (100.0 mg, 0.150 mmol) in CH_2Cl_2 (10 mL) under a CO atmosphere for 16 h to give compound **2j** as a yellow oil. Yield: 37 mg, 0.106 mmol, 35%. Compound **2j** was characterized by NMR methods as a mixture of rotamers in 3:1 molar ratio.

NMR data. Major isomer: ^1H NMR (300 MHz, $CDCl_3$, 258 K) δ 7.70 (dd, *J* = 7.7, 1.5 Hz, C_6H_4), 7.37 (td, *J* = 7.6, 1.7 Hz, C_6H_4), 7.31 (td, *J* = 7.5, 1.5 Hz, C_6H_4), 7.16 (dd, *J* = 7.5, 1.5 Hz, C_6H_4), 3.86 (s, CH), 3.81 (m, NCH_2), 3.66 (s, OCH_3), 3.16 (m, NCH_2), 2.94 (m, NCH_2), 2.20 (s, NMe_2), 1.70–1.59 (m, NBu), 1.59–1.47 (m, NBu), 1.46–1.30 (m, NBu), 1.18–0.97 (m, NBu), 0.94 (m, CH_3), 0.75 (m, CH_3); $^{13}C\{^1H\}$ NMR (75 MHz, $CDCl_3$) δ 172.30 (s, CO), 170.15 (s, CO), 138.11 (s, C), 133.27 (s, C), 129.20 (s, CH), 128.62 (s, CH), 128.23 (s, CH), 125.97 (s, CH), 71.11 (s, CH), 52.12 (s, OCH_3), 48.71 (s, NCH_2), 44.26 (s, NCH_2), 43.81 (s, NMe_2), 30.59 (s, CH_2), 29.47 (s, CH_2), 20.50 (s, $\underline{C}H_2Me$), 20.06 (s, $\underline{C}H_2Me$), 14.04 (s, CH_3), 13.73 (s, CH_3). *Minor isomer*: ^1H NMR (300 MHz, $CDCl_3$, 258 K) δ 7.50 (dd, *J* = 7.3, 1.8 Hz, C_6H_4), 7.43–

7.25 (m, C_6H_4), 7.17 (m, C_6H_4), 4.32 (s, CH), 3.80 (m, NCH_2), 3.66 (s, OCH_3), 3.15 (m, NCH_2), 3.03–2.86 (m, NCH_2), 2.23 (s, NMe_2), 1.72–1.29 (m, NBu), 1.18–0.97 (m, NBu), 0.93 (m, CH_3), 0.76 (m, CH_3); IR (ν, cm^{-1}): 1735 (COO), 1623 (CON); ESIMS (positive mode) (*m*/*z*): 349.0 $[M + H]^+$; anal. Calcd for $C_{20}H_{32}N_2O_3$ (348.24): C, 68.93; H, 9.26; N, 8.04; found: C, 69.06; H, 9.35; N, 8.10.
